# Analgesic and clinical side effects of paracetamol and meloxicam in female dogs after elective ovariectomy or ovariohysterectomy – randomized clinical trial

**DOI:** 10.1186/s13028-026-00892-3

**Published:** 2026-11-10

**Authors:** Kati Salla, Vuokko Pekkola, Ira Kallio-Kujala, Jaan Lepajoe, Daniela Casoni

**Affiliations:** 1 Department of Equine and Small Animal Medicine, Faculty of Veterinary Medicine, University of Helsinki, P.O. Box 57, Helsinki, FI-00014 Finland; 2 Experimental Surgery Facility, Experimental Animal Centre, University of Bern, Freiburgstrasse 15, Bern, 3010 Switzerland

**Keywords:** GCMPS-SF, NSAID, Ovariohysterectomy, Post-surgical pain, Von Frey filaments

## Abstract

**Background:**

This prospective, randomized, blinded, controlled clinical trial aimed at comparing the analgesic and antihyperalgesic effect of paracetamol and meloxicam following elective ovariectomy or ovariohysterectomy in healthy female dogs. In addition, it aimed at evaluating gastrointestinal side effects up to 48 h after surgery. Standardised anaesthesia protocol was utilised in all dogs and, at the end of the surgery, either meloxicam (0.2 mg/kg, Group M, *n* = 15 dogs) or paracetamol (10 mg/kg, Group P, *n* = 15 dogs) was administered intravenously. Pain was evaluated at 1, 2, 3, 4 and 6 h after the administration of either paracetamol or meloxicam using short form of Glasgow composite pain scale (GCPS-SF). Rescue analgesia with intravenous methadone (0.2 mg/kg) was administered in case of ≥ 6 over 24 points. The success rate of each treatment was calculated based on the need of rescue analgesia within 6 h after surgery. Subsequently, the success rate was assessed for non-inferiority between treatments. The non-inferiority margin was set as -33%. Mechanical nociceptive thresholds and sensitivity to Von Frey filaments were tested at 2, 4 and 6 h after the administration either paracetamol or meloxicam. Once discharged, animals were remotely evaluated through a standardized questionnaire.

**Results:**

Rescue analgesia was administered at the hospital during the 6-hour post-operative period in a total of 15 dogs without difference between groups (*p* = 0.358). The success rate for analgesia was (9/15) 60% and (6/15) 40% in group M and P, respectively. The lower limit of the 95% CI of the difference for success rate (-55%, 15.5%) was lower than the margin set for non-inferiority (-33%) and therefore the non-inferiority of the group P versus group M was not confirmed. Mechanical sensitivity increased in both group after surgery. Mild self-limiting gastrointestinal side effects were detected in both groups.

**Conclusions:**

Dogs receiving paracetamol needed more rescue analgesia than dogs receiving meloxicam in the post-operative period. The rate of success with both drugs was lower than expected. Frequent assessment of pain is mandatory to ensure efficient analgesia also beyond the first post-operative day and future studies are warranted to validate methods for out-of-hospital pain assessment.

**Supplementary Information:**

The online version contains supplementary material available at https://doi.org/10.1186/s13028-026-00892-3.

## Background

Non-steroidal anti-inflammatory drugs (NSAIDs) are a mainstay for the management of acute inflammatory pain in veterinary medicine. Meloxicam is a preferential COX-2 inhibitor NSAID, widely used in dogs. It has been reported that preoperative administration of NSAIDs brings about higher efficacy if compared with its post-operative use [1]. Meloxicam was demonstrated not causing acute kidney failure in healthy experimental dogs experiencing intraoperative hypotension [2, 3]. However, the risk of kidney damage associated with potential systemic hypotension and decrease of renal blood flow during inhalant anaesthesia might still justify the recommendation to use them post-operatively in clinical patients undergoing surgery [4]. Furthermore, it has been demonstrated that the plasma disposition of meloxicam can be altered by anaesthesia and surgery leading to decrease in the volume of distribution, increasing initial plasma peak concentration and might increase the risk of concentration-dependent adverse effects [5]. A balanced approach to prevent and treat nociception and pain, i.e. the combination of different classes of analgesic drugs and techniques is therefore preferred in clinical situations [4].

Alternatives to NSAID administration in the post-operative period are still desired for patients with gastro-intestinal or renal diseases, and in emergency settings where analgesia must often be provided to patients at higher risk of acute kidney injury [4]. In the last decade, the use of paracetamol has become increasingly widespread among practitioners and has been identified by some authors [6] as useful alternative to classical NSAIDs, when concerns about their side effects exist. In a more recent report, paracetamol has been shown to be widely accepted among UK veterinarians, especially as part of multimodal analgesia or when NSAIDs are contraindicated [7]. The mechanism of action of paracetamol appears to be multiple and not completely elucidated, yet it seems mainly acting through three pathways [8, 9]: (1) prostaglandin inhibition through peroxidase inhibition and therefore inhibition of both COX 1 and 2 (2) activation of descending inhibitory serotoninergic pathways (3) endocannabinoids enhancement. Despite its widespread clinical use, literature regarding efficacy of paracetamol at clinically relevant doses in dogs is still scarce and partially contradictory. Paracetamol up to 15 mg/kg three times per day are reported to be well tolerated in dogs [10]. Orally administered combination of paracetamol and codeine has been demonstrated non-inferior to meloxicam for postoperative analgesia in dogs when used alongside methadone and buprenorphine [11]. Paracetamol (15 mg/kg IV) provided comparable pain relief to meloxicam and carprofen in dogs undergoing ovariohysterectomy [12]. In contrast, intravenous paracetamol (20 mg/kg) did not provide significantly better post-operative analgesia in dogs undergoing ovariohysterectomy and receiving pre-operative analgesic meperidine, when compared to saline [13].

Pain is a complex and subjective experience, in which sensory and affective (or emotional) components can be recognized and separately assessed [14]. The use of quantitative sensory testing (QST) can provide a quantitative assessment of the somato-sensory component of pain and objectivize hyperalgesia and allodynia [15]. In particular, measuring mechanical sensory thresholds with von Frey anesthesiometers and pressure algometers is deemed feasible in dogs and generating reliable results [16]. The emotional component of pain is currently mainly evaluated in non-verbal patients through behavioural changes over time, both in terms of modification of normal behaviour and development of abnormal behaviours [17]. The Glasgow composite measure pain scale is a multi-item behavioural pain assessment tool, to measure acute pain in dogs. Its short form (GCPS-SF) has been validated for the assessment of acute post-operative pain in clinical contexts. Importantly, the score is linked to an intervention level, which guides the requirement for additional analgesia [18, 19].

The primary aim of the study was to compare the analgesic and anti-hyperalgesic effects of paracetamol and meloxicam in the first 6 post-operative hours following elective ovariectomy/ovariohysterectomy in healthy female dogs. The secondary aim was to assess analgesic and gastrointestinal side effects up to 48 h after the surgery. The null hypothesis was that the rate of success of paracetamol, quantified as no need of rescue analgesia during 6 post-operative hours, was non-inferior to meloxicam.

## Methods

This trial was conducted in clients-owned dogs, admitted to Veterinary Teaching Hospital, Faculty of Veterinary Medicine, University of Helsinki, Finland after owners’ spontaneous contact with the structure. The Animal Experiment Board of Finland (ESAVI/8506/2020) and the Finnish Medicines Agency approved the study. A written consent was obtained from owners, prior starting data collection. This study is reported according to the CONSORT guidelines (Additional file 1). Healthy female dogs classified as ASA 1 and 2, older than 6 months and younger than 12 years undergoing elective ovariectomies or ovariohysterectomies were included in the study. Dogs were considered healthy on the base of clinical exam, normal haematology and hepatic and renal values (ALT, AST, ALP, creatinine, urea). Dogs receiving other concurrent medications, or that have received either paracetamol, meloxicam or other NSAIDs in the last 48 h prior the scheduled surgery, and dogs with BCS ≥ 4/5 were excluded from the study. To verify the non-inferiority of paracetamol, stating equivalence for percentage of success differing no more than 33% [20] and assuming 90% success rate for both treatments [11], with 80% power, and 5% alfa error, a minimum of eleven dogs per group was needed.

### Study design

In this clinical prospective randomized controlled study, thirty dogs were recruited to account for a potential loss during the study. Dogs were randomly allocated with a 1:1 ratio to either paracetamol group (group P) or meloxicam group (group M). Randomization was performed using a dedicated software (www.randomization.com). The investigator responsible for data collection (KS) was blinded to the treatment.

**Fig. 1:**
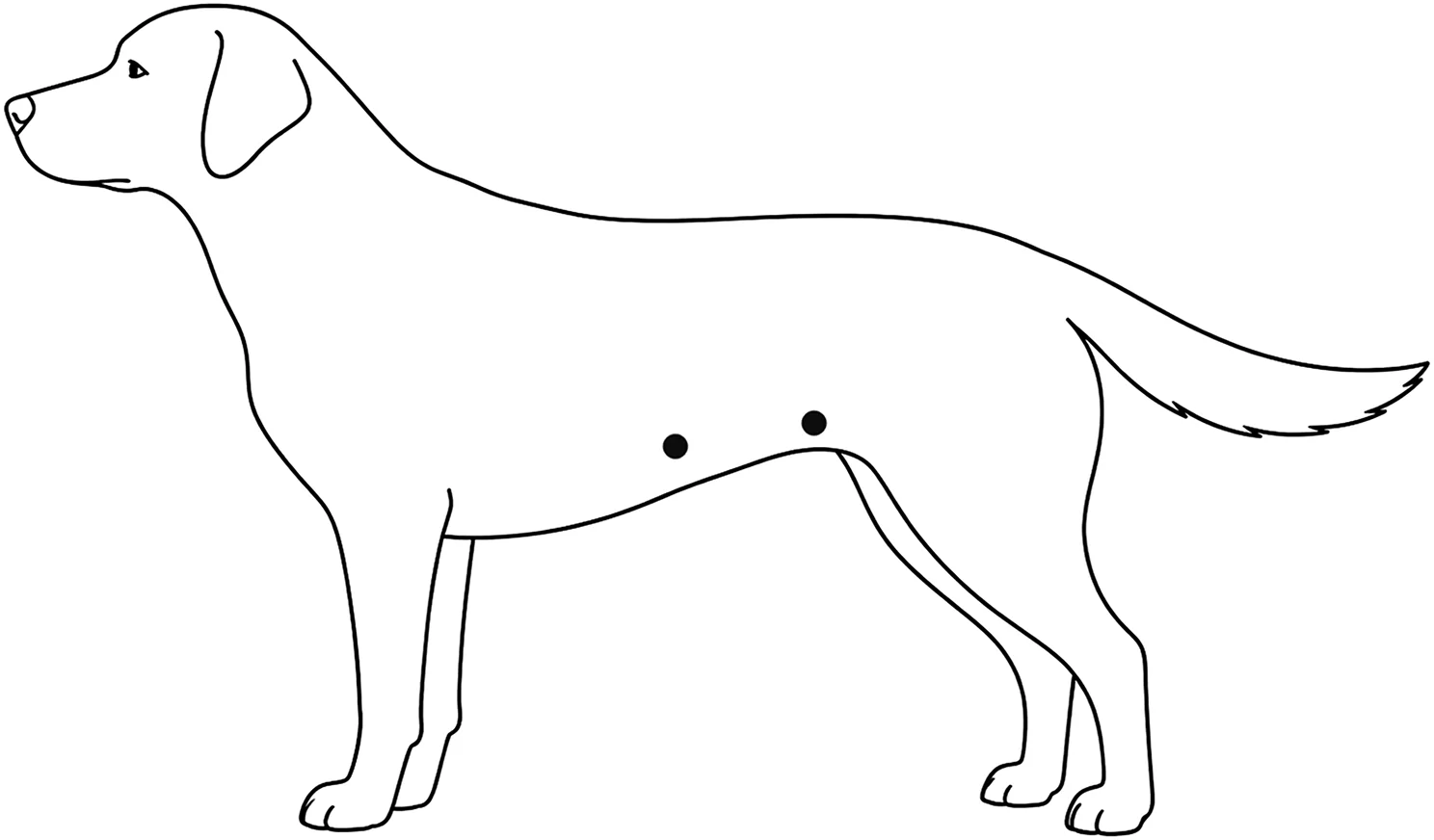
von Frey and pressure algometer stimulation points. Two points per ventrolateral abdominal wall (black dot) that were selected for von Frey filament and pressure algometer stimulations

In the morning of the surgery, the baseline (BL) pain assessment using short form of Glasgow composite pain scale (GCPS-SF) [18], and the evaluation of the tactile sensitivity and mechanical nociceptive threshold was performed. Tactile sensitivity was assessed with Von Frey filaments (Aesthesio^®^ Tactile Sensory Evaluators, DanMic Global, LLC, San Jose, CA, USA) using a set of twenty von Frey monofilaments ranging from 0.008 to 300 g of target force (corresponding 2.53–292 g/mm^2^) [21, 22]. Briefly, the dogs were gently restrained on standing position, and once the dogs were comfortable in this position, two points per ventrolateral abdominal wall (Fig. 1) at the level of cranial and caudal end of the estimated surgical wound were selected and stimulated starting with the thinnest monofilament. In absence of response, the next monofilament was tried until an escape from the filament or avoidance response was observed. Once the dog showed a reaction, the assessment at that point was ceased. Prior to statistical analysis reactions to von Frey fibres were coded from 0 (target force of the filament < 1 g) to 8 (no response to the target force of 300 g) (Additional file 2.) Mechanical thresholds were measured after assessment of tactile sensitivity using a mechanical pressure algometer with a rubber tip area of 1 cm^2^ (FDN 100–Algometer, Wagner Instruments, USA). The algometer was regularly calibrated according to the manufacturer’s instructions. After placing the tip of the algometer on the selected points, the force was applied at an increasing rate of approximately 2 N/s from 10 N (lower detection limit) until the dog exhibited an avoidance response or the applied force reached 70 N (cut-off). A threshold of 8 N was assigned to assessments in which dogs showed an avoidance response at contact with the tip of the algometer. Every assessment of tactile sensitivity and mechanical pressure threshold was repeated twice per point at approximately 30 s intervals.

Dogs were premedicated with 0.02 mg/kg of acepromazine (Plegicil, Pharmaxim, Sweden) and 0.2 mg/kg of methadone (Insistor, VetViva Richter, Austria 0.2 mg/kg) injected intramuscularly. After 30 min, general anaesthesia was induced with IV propofol (PropoVet 10 mg mL^− 1^, Abbott Laboratories Ltd, UK) titrated to effect (1–4 mg/kg). Maintenance of general anaesthesia was carried out with sevoflurane in oxygen. During anaesthesia HR, SpO_2_, non-invasive oscillometric blood pressure, oesophageal temperature, (Life Scope TR; Nihon Kohden), respiratory rate, expired fraction of CO_2_ (ETCO_2_) and sevoflurane (ET_Sevo_) (Dräger Perseus A500; OneMed Ltd, Finland) were monitored continuously and recorded every 5 min until the end of the surgery. The depth of anaesthesia was monitored with 5-minute intervals based on clinical signs (e.g., palpebral reflex, eye position and jaw tone). An incisional line block along the linea alba was performed with 2 mg/kg of ropivacaine 0.5% (Ropivacain Fresenius Kabi 5 mg/mL, Fresenius Kabi, Sweden) after surgical preparation and before surgical draping. In addition, over each ovarian suspensory ligament, 2 mg/kg of lidocaine 2% (Lidor, VetViva Richter, Austria) was splashed approximately one minute before the ovary was manipulated. Every surgery was carried out by senior surgeons supported by undergraduate veterinary students. The decision whether performing hystherectomy was in the hands of the senior surgeon and recorded as yes/no. Intraoperative nociception was evaluated through autonomic variables; baseline values were recorded immediately before skin incision. If at least two out of three parameters among mean blood pressure, heart rate and respiratory rate were increased of 20% from baseline recordings in response to a nociceptive stimulus, intravenous fentanyl was administered at 3 µg/kg (Fentanyl-Hameln 50 µg mL-1, Hameln Pharma Plus Gmbh, Germany). The need of fentanyl during the surgery was recorded as yes/no, and the total dosage of fentanyl recorded. At the time of suturing the linea alba, either IV meloxicam 0.2 mg/kg (Metacam 5 mg mL^− 1^, Vetcare Ltd, Salo, Finland) (group M) or IV paracetamol 10 mg/kg (Paracetamol B. Braun 10 mg/ml, B. Braun Melsungen AG, 34209 Melsungen, Germany) (group P) was administered. The duration of general anaesthesia was recorded.

Dogs were recovered from general anaesthesia and kept in the recovery room under researcher supervision for at least six hours after recovery from general anaesthesia. Pain was evaluated at 1, 2, 3, 4 and 6 h after the administration of either paracetamol or meloxicam using GCPS-SF. The dogs were evaluated in the recovery room, by the same operator (KS) who tested they baseline thresholds before anaesthesia. In addition, the thresholds response to Von Frey filaments and mechanical algometer were recorded at 2, 4 and 6 h after the IV administration of either paracetamol or meloxicam. Intravenous methadone (0.2 mg/kg) was administered as a rescue analgesia if GCPS-SF scored ≥ 6 over 24 points. The need of methadone was recorded as yes/no. The success rate of each treatment was calculated based on the need of rescue analgesia within 6 h after surgery. In particular, the treatment was considered successful in all the cases in which no rescue analgesia was administered.

After discharging the dogs, 0.1 mg/kg of meloxicam PO (once per day) in group M or 10 mg/kg of paracetamol PO (three times per day) in group P were prescribed for 5 days after surgery. The owner received prescriptions in opaque envelopes covered with label indicating “primary analgesia” and “drug for rescue analgesia” to ensure blinding of researcher (KS). The owners were instructed to administer primary analgesia to their dogs and to add rescue analgesia in case they recognised signs of pain in the period of time between discharge from the hospital and contact with the researcher. The envelope contained also the exact instructions on the frequency of administration of the drugs. On the day after surgery (POD1) and on the following day (POD2), the owners were contacted by phone (KS) and they were asked questions (Additional file 3.) related to the general wellbeing of the dogs, and clinical signs possibly representing side effects of analgesics. Furthermore, the owners were asked to evaluate the pain of their dogs by answering questions formulated using a modified version of the GCPS-SF excluding the part IV and VI, which resulted in a maximum sum of 15 points (Additional file 4.). The administration of rescue analgesia at home was recorded as yes/no. The rescue analgesia (meloxicam in group P and paracetamol in group M) was implemented if analgesia was deemed insufficient based on the interview at POD1(≥ 4/15 points i.e. 27% of maximum score). The owners were instructed in this case to administer the rescue drug according to the instruction written in the prescription for rescue analgesia. In case of unexpected event of severe pain or side effects, such as severe gastrointestinal disorders, the owners were encouraged to reach the researchers spontaneously, so that the dogs could be re-evaluated at the hospital.

### Statistical analysis

If methadone was administered as rescue analgesia postoperatively, the following GCPS-SF, algometer and von Frey recordings were excluded from the analysis. The mean value of the measured mechanical thresholds and the value of the thinnest filament of von Frey at which a response was evocated at a predetermined time point were used for statistical analyses and compared between the groups. Data were analysed with SPSS software, version 27 (IBM SPSS Statistics, IBM Corp., Armonk, NY, USA). The normality of data distribution was evaluated with the Shapiro-Wilk test. Repeatedly measured algometry data was analysed by means of mixed-model analysis of variance with post-hoc Bonferroni adjustment at selected time points. Parametric data measured only once were compared using unpaired two-tailed t-tests. Categorical variables were compared between the groups with the Mann-Whitney U test. For categorical variables recorded as yes/no, Fisher’s Exact test was applied. The relative risk that a clinical side effect appeared in group P versus group M was calculated and the significance verified with the Fisher’s Exact test. Kaplan-Meier survival curves were generated to estimate the time to reach the cut-off value for GCPS-SF. The Log-Rank test was used to assess whether there were significant differences in survival between the groups. Individual dogs that had baseline mechanical threshold lower than a mean minus SD value from all dogs was recorded yes/no. The correlation between low baseline mechanical threshold and the need of rescue analgesia with methadone was assessed using Pearson correlation test. Non-inferiority of P to M was claimed if the lower limit of the 95% of confidence interval (CI) for the difference in success rate was greater than − 33% [20]. This test for non-inferiority was only performed for the primary outcome variable (success rate at 6 h) if superiority was not demonstrated between the groups; all other variables were tested for the superiority of group M versus group P. Data are presented as mean ± standard deviation (SD) or median (minimum–maximum), as appropriate. A P-value below 0.05 was considered statistically significant.

## Results

Data collected from 30 dogs were included for analysis, of which 15 belonged to M and 15 to P group. Mean body weight was significantly higher in group P (18.9 ± 13.8 kg) than in group M (10.6 ± 6.5 kg) (*P* = 0.049). The age of dogs in group P was 3.9 ± 2.3 years and in group M 3.0 ± 2.1 years (*P* = 0.284). Surgeries were successfully completed in each dog, and ovariohysterectomy was done to three and six dogs in group P and group M, respectively (*P* = 0.427). The duration of the general anaesthesia was 2.6 ± 0.2 h and 2.7 ± 0.3 h in group P and M, respectively (*P* = 0.285). The need of intraoperative fentanyl was not different between groups, as 6/15 dogs in group P and 7/15 dogs in group M received fentanyl during the surgery (*P* = 1.000). The total dosage of fentanyl was 1.6 ± 3.2 µg/kg in group P and 1.9 ± 3.4 µg/kg in group M (*P* = 0.779).

**Fig. 2:**
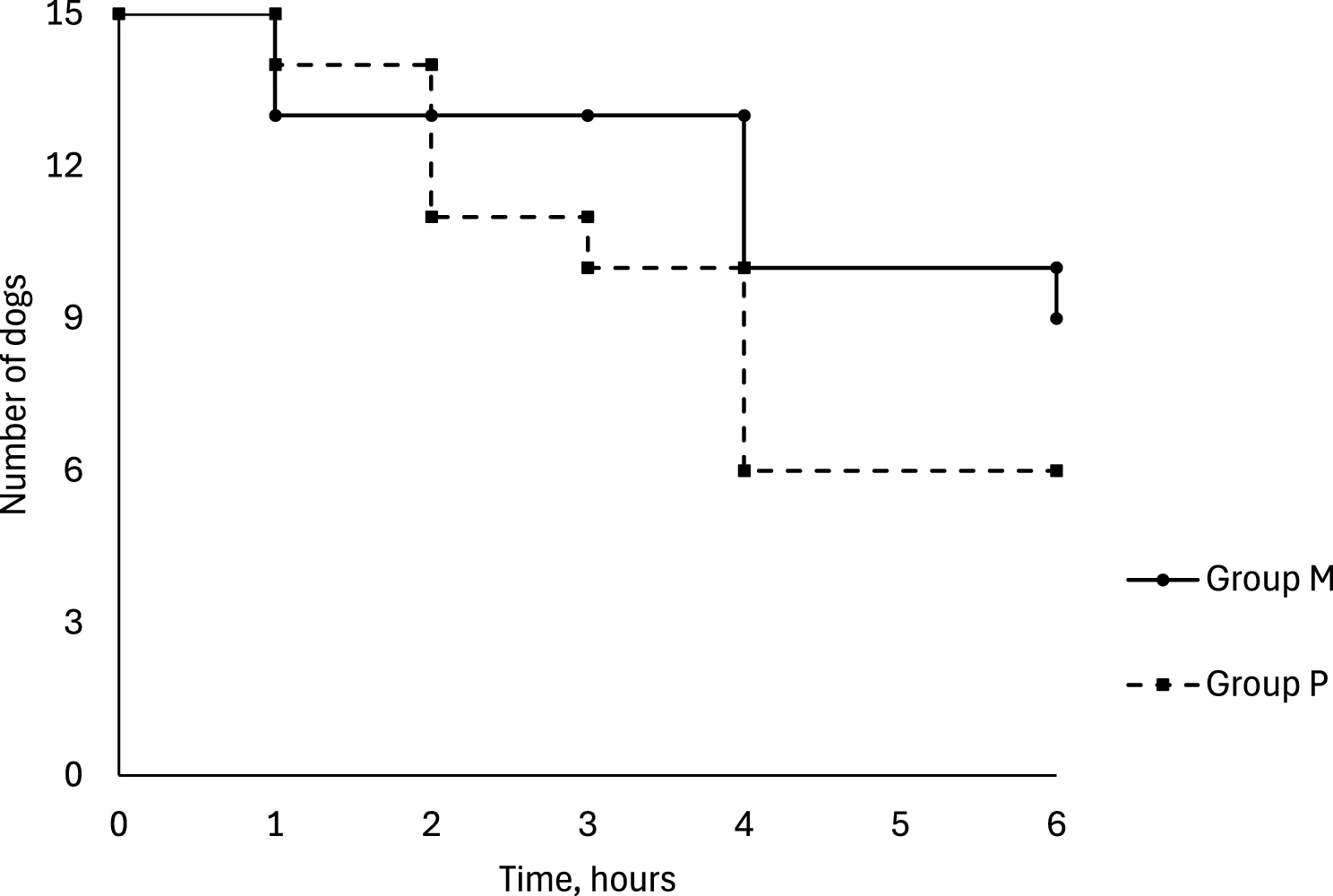
The time of rescue analgesia after surgery. Kaplan Meier curve presenting time when dogs medicated with either meloxicam 0.2 mg/kg IV (group M, n=15) or paracetamol 10 mg/kg IV (group P, n=15) reached the cut-off value (composite Glascow Pain Scale-short form ≥ 6 over 24 points) for administering of rescue analgesia

Methadone as rescue analgesia was administered (GCPS-SF was ≥ 6 over 24 points) at the hospital during the 6-hour post-operative period in a total of 15 dogs. The need of methadone in general (yes/no) was not different between groups (*P* = 0.358). The success rate for analgesia was (9/15) 60% and (6/15) 40% in group M and P, respectively. The lower limit of the 95% CI of the difference for success rate (-55%, 15.1%) was lower than the margin set for non-inferiority (-33%), therefore non-inferiority of the group P versus group M could not be confirmed. The median time of administering the first dose of methadone was 3 h (1–4) and 4 (1–6) in group P and group M, respectively (*P* = 0.689). Figure 2. presents the times to reach the cut-off value for GCPS-SF. The Log-Rank (Mantel-Cox) test did not reveal a significant difference in survival distributions between the groups (χ² = 1.369, *P* = 0.242). However, the total number of methadone boluses was significantly higher in group P (15 boluses) than in group M (7 boluses) (*P* = 0.026).

The numerical results of GCPS-SF, mechanical thresholds and tactile sensitivity are presented in Table 1. Baseline measurements of GCPS-SF, mechanical threshold or tactile sensitivity were not different between groups. Moreover, the number of dogs that had low baseline mechanical threshold was 3/15 and 5/15 dogs in group P and group M, respectively (*P* = 0.341). There was no difference in GCPS-SF over 6 h post-operative period between groups. Mechanical thresholds were lower in group M in comparison to group P at every post-operative evaluation, but significance was reached only at 4 h timepoint with recordings of 19.2 ± 5.1 N and 12.9 ± 6.3 N in group P and M, respectively (*P* = 0.045). In addition, the most sensitive recordings with von Frey fibres were 0.6 g in group M and 4 g in group P, and the tactile sensitivity was higher in group M compared to group P at 2 h timepoint (*P* = 0.033). There was a weak positive correlation (Pearson correlation value = 0.262) between the low baseline mechanical threshold and the need of rescue analgesia with methadone.

**Table 1:** Results of Glasgow composite pain scale, tactile sensitivity score and mechanical pressure thresholds

	Group	Baseline *n* = 15(P) *n* = 15 (M)	1 h *n* = 15 (P) *n* = 15 (M)	2 h *n* = 14 (P) *n* = 13 (M)	3 h *n* = 11 (P) *n* = 13 (M)	4 h *n* = 10 (P) *n* = 13 (M)	6 h *n* = 6 (P) *n* = 10 (M)
GCPS-SF	*P*	0 (0–0)	4 (1–6)	4 (1–11)	4 (2–6)	3.5 (2–9)	3 (1–5)
	M	0 (0–0)	5 (2–8)	4 (2–5)	4 (2–5)	3 (1–6)	3 (1–6)
P-value		1.000	0.313	0.320	0.388	0.418	0.725
Tactile sensitivity score	P	4 (2–8)	NA	4.5 (2–8)	NA	6 (1–8)	6 (1–8)
	M	3 (2–8)	NA	4 (0–5)	NA	3 (0–8)	3 (1–6)
P-value		0.567	NA	0.033	NA	0.167	0.152
Mechanical pressure threshold	P	26.6 ± 6.3	NA	22.0 ± 8.6	NA	19.2 ± 5.1	16.5 ± 7.8
	M	23.4 ± 7.2	NA	15.7 ± 6.6	NA	12.9 ± 6.3	11.3 ± 4.6
P-value		0.354	NA	0.098	NA	0.045	0.091

Median (max-min) of Glasgow composite pain scale (GCPS-SF) and tactile sensitivity score (see coding of target forces of filaments in additional file 1) and mean ± SD of mechanical pressure thresholds in dogs at baseline and during 6 post-operative hours when administered either meloxicam 0.2 mg/kg IV (Group M) or paracetamol 10 mg/kg IV (Group P) at the end of ovariectomy/ovariohysterectomy. P-values refer to comparions between groups at timepoints and *P* < 0.05 was considered statistically significant

Three dogs that received rescue analgesia on POD 1 based on pain estimated by the owners before the interview with the researcher (KS), all belonging to the group P. According to interview on POD1, eleven dogs had modified GCPS-SF ≥ 4/15 points, and five of the belonging to group P and seven group M. The total number of dogs that received rescue analgesia on POD2 was 12, and nine of them belonging to group P (*P* = 0.030). However, the pain score assigned by the owner did not differ among groups in either day. In particular, the modified GCPS-SF on POD1 was 3 (2–4) in group M and 3 (2–4) in group P (*P* = 0.410). The modified GCPS-SF on POD2 was 2 (0–3) in group M and 2 (0–2) in group P (*P* = 1.000).

The total number of individual dogs that needed any rescue analgesia either in hospital or at home was 13 out of 15 dogs in group P, and in eight out of 15 dogs in group M (*P* = 0.054). This resulted an overall analgesic success rate of 13% and 47% in group P and group M, respectively.

The clinical side effects noticed by the owners after surgery and their likelihood are reported in Tables 2 and 3. In both groups, their frequency decreased over time. No dog needed to be re-admitted to the hospital to handle these side effects. No significant differences in the likelihood that a clinical sign was present between the two groups was identified.

**Table 2:** Clinical side effects on the first day after surgery

Drooling	Group *P*	Group M	Relative Risk		*P*-value
	2/15	3/15		1.08	1
Dysorexia/anorexia	12/15	8/15		1.5	0.24
Polydipsia/oligodipsia	10/15	9/15		1.11	1
Vomiting	5/15	1/15		0.714	0.17
Poliuria/oliguria	2/15	0/15		0.87	0.48
Loose stool	3/15	3/15		1	1

Clinical side effects reported by the owners in the first postoperative day in dogs treated with 0.2 mg/kg meloxicam SID (Group M, *n* = 15 dogs) or with 10 mg/kg paracetamol TID (Group P, *n* = 15 dogs) after surgical ovariectomy /ovariohysterectomy. The number of dogs reporting the side effects are indicating as dogs affected/number of dogs per group

**Table 3:** Clinical side effects on the second day after surgery

	Group *P*	Group M	Relative Risk	*p*-value
Drooling	0/15	0/15	-	-
Dysorexia/anorexia	4/15	0/15	0.1	0.1
Polydipsia/oligodipsia	4/15	3/15	0.92	1
Vomiting	4/15	1/15	0.33	0.33
Polyuria/oliguria	0/15	0/15	-	-
Loose stool	5/15	1/15	5	0.169

Clinical side effects reported by the owners in the second postoperative day in dogs treated with 0.2 mg/kg meloxicam SID (Group M, *n* = 15 dogs) or with 10 mg/kg paracetamol TID (Group P, *n* = 15 dogs) after surgical ovariectomy /ovariohysterectomy. The number of dogs reporting the side effects are indicating as dogs affected/number of dogs per group

## Discussion

The results of this study do not support the hypothesis that paracetamol, at a dose of 10 mg/kg IV, would provide a non-inferior rate of analgesic success than meloxicam at the dose of 0.2 mg/kg IV. This finding contrasts with two previous studies conducted in dogs. The combination of paracetamol/codeine was not inferior to meloxicam when a routine administration of buprenorphine was provided during first 24 h after various surgeries [11]. Similarly, Hernández-Avalos et al., demonstrated that paracetamol administered before surgical incision was not inferior to meloxicam when additional intraoperative analgesia with constant rate infusion of fentanyl was provided during ovariohysterectomy [12]. However, when paracetamol was administered in patients receiving meperidine as sole pre- and intraoperative analgesic no detectable analgesia was recorded [13]. These findings seem to suggest that paracetamol counteracts effectively pain only if integrated in a strategy of multimodal analgesia which centres on intraoperative or post-operative use of opioids. In our study, opioids were administered preventively and, afterwards, only as rescue treatment. The success rate of our study was lower in comparison to the earlier study of Hernandez-Avalos et al. [12] where 90% of success rate in post-operative analgesia was achieved with both meloxicam (0.2 mg/kg IV) and paracetamol (15 mg/kg IV) in dogs undergoing ovariohysterectomy. The low success rate observed in both groups in our study may have several explanations. It could be an effect of the timing of drugs’ administration, as both meloxicam and paracetamol were administered at the end of the surgery, or an effect of the individualised intra-operative opioids administration with no routine opioid administered during or after surgery. It must also be highlighted that the dose of paracetamol was higher, and the duration of anaesthesia and surgery was shorter in the study of Hernandez-Avalos et al. [12] than in the present study. Although the duration of surgery does not seem to be correlated with the intensity of post-operative pain per se [23], the average duration of our surgeries in a teaching hospital was appreciably long. A longer surgery might imply a protracted tissue stimulation and inflammation, affecting the efficacy and the duration of post-operative analgesia. Furthermore, considering both the short half-life of methadone and the duration of its analgesic effects [24] it is unclear whether, upon recovering from general anaesthesia, the animals were still able to benefit from its action. Within the dose range of 10 to 15 mg/kg [7], we selected a dose of paracetamol of 10 mg/kg. We cannot therefore rule out the possibility that, had we used higher doses, the efficacy would have been greater. It should be also appreciated that use of different methods to assess pain might have resulted in different outcomes between studies. Indeed, while in our study GPCS-SF and quantitative sensory testing were used, Dynamic Inter active Visual Analog Scale (DIVAS) and Pain Scale of the University of Melbourne (UMPS) scoring systems was used by Hernandez-Avalos et al. [12]. In our study we selected the margin of non-inferiority according to the he definition of clinically important difference in pain assessment measures in humans [20]. This selection of margin was supported by the authors experience about the clinically relevant difference in the rate of success. Fixed margin approach using a proven effect size obtained from meta-analysis was recently suggested by Santhanna & Joshi [35]. However, the absence of metanalysis as well as of studies with large sample size would have precluded in our case the use of this approach.

Ovariohysterectomy is a painful procedure and despite multimodal analgesia, careful post-operative assessment is mandatory in order to avoid pain. In general, when a surgery is performed, the first 24 h are considered to be the most painful period after surgery [24–26]. In the present study, the highest frequency of post-operative scores exceeding the intervention threshold for rescue analgesia was recorded in the time window of 3–4 h after the end of anaesthesia in both groups. This finding is in line with Quarterone et al. [27] and del Mar Granados [28], which concluded in their independent studies that regular postoperative pain assessments after ovariohysterectomy are necessary for at least 4 h in dogs to ensure proper postoperative pain diagnosis and management. Although the need for rescue analgesia was not different between group M and group P, the number of rescue analgesia boluses during the first six postoperative hours was greater in group P than in group M. This could be due to differences in the pharmacokinetic properties of meloxicam and paracetamol, such as the recommended dosing intervals 24 and 8 h for meloxicam [29] and paracetamol [28], respectively. Recently, plasma paracetamol concentrations between 10.0 and 20.0 µg/mL has been demonstrated to be associate with analgesic effects, assessed with GCPS-SF and mechanical nociceptive thresholds, in client owned dogs [28]. In the study of Leung et al. [13], no plasma concentration above 10 µg/mL were measured in any client owned dog at 80 min from the intravenous administration of 20 mg/kg paracetamol. However, plasma drug concentrations were not measured in the present study, thus it cannot be distinguished whether the more frequent administration of rescue analgesia in group P was due to poor analgesic effects of paracetamol per se, or due to inappropriate dosage associated with low plasma drug concentration over time.

Quantitative sensory testing showed higher mechanical sensitivity in group M than in group P. It should be kept in mind that pain is a complex individual experience, and mechanical hyperalgesia and allodynia do not equal the level of pain perception, neither represent pain in its entirety. Sanchis-Mora et al. [30] demonstrated that small dogs have lower mechanical thresholds than larger dogs. In the present study, the weight of dogs was not equal between groups. This result was unexpected and could have been avoided by using block design with stratification in different weight classes to ensure even distribution among groups. However, similar baseline thresholds were recorded in both groups, independently of animals’ weight. Therefore, the actual impact of the size of the dogs in determining our results is unknown. Tactile and mechanical sensitivity was tendentially lower in group P than M. In experimental rats, it was demonstrated that paracetamol, but not metamizole could reduce mechanical sensitivity in a model of inflammatory pain both in the affected and non-affected leg [31]. Interestingly, the systemic administration of naltrexone, antagonised the beneficial effect of paracetamol, underlying its multiple mechanism of action beyond the pure COX inhibition [31]. A similar effect could be speculated in our case, but data on dogs, to the best of Author’s knowledge, are missing. In our cohort, there was a weak correlation between animals with low baseline mechanical thresholds and the need of rescue analgesia, independently from the group. The predictive value of low mechanical thresholds must be verified in larger cohorts of dogs.

Multimodal preventive analgesic approach was applied, including premedication with long-acting opioid, and administration of topical and peri-incisional locoregional anaesthetics, as we evaluated the efficacy of paracetamol in a clinically relevant setting. In earlier studies investigating the analgesic efficacy of paracetamol for post-operative pain in dogs, locoregional anaesthetic techniques were not applied [11–13]. Despite the multimodal approach applied in the present study, nearly half of dogs needed intraoperative rescue analgesia in both groups. This indicates that our initial approach did not guarantee intraoperative antinociception in all patients. However, deficits of antinociception could be successfully corrected by administration of rescue opioid in all cases. Neither the relation between intraoperative antinociception and post-operative pain nor the relation between intraoperative nociception and peripheral sensitization is straightforward and clear. Although still largely utilised, the concept of “pre-emptive” or “preventive” analgesia to reduce peripheral and central sensitization has overall unfortunately proven less efficacy than expected [32, 33]. According to our results, it might be stated that neither meloxicam nor paracetamol administered at the time of abdominal closure in a protocol of balanced analgesia were efficient in preventing allodynia, hyperalgesia or behavioural signs of pain in every ovariohysterectomized dog.

To the author’s knowledge this is the first study reporting the effects of paracetamol or meloxicam on pain related behaviours and overall wellbeing of dogs over 48 h, when the dogs are in their familiar environment e.g. at home. We used a structured questionnaire that included questions related to overall wellbeing and modified GCPS-SF, although the questionnaire was not previously validated. Regarding pain assessment and the need of rescue analgesia at home, a similar pattern as the one observed in early post-operative period in hospital was observed. Dogs in group P needed more rescue analgesia than in group M. Previous study in dogs undergoing ovariohysterectomy demonstrated overt behavioural changes after surgery and suggested a minimum of three days’ pain relief after ovariohysterectomy [34]. Our results highlight the need of an individualised approach as post-operative pain may not be acceptably managed with single analgesic agent in some dogs, whereas inappropriate polypharmacy could lead to unwanted side effects in other. In particular, we identified the need for validated species-specific owners-friendly tools for acute pain assessment. Indeed, the recommendation of administering rescue analgesia based on subjective pain assessment might have led to overtreatment in some patients, due to overinterpretation of some less specific signs. The overall wellbeing of dogs did not differ between groups on POD1 and POD2. The most common reported side effects were related to nausea (drooling, vomiting) and changes in appetite. Interestingly, the gastro-intestinal signs appeared also in the P group. However, since the dogs received as rescue analgesia either paracetamol or meloxicam several dogs were administered with both drugs at POD1 or POD2. Based on our results, it is not clear if these findings were related to the stress of general anaesthesia and surgery, the drug used, their combination, the presence of visceral pain or the combination of all these factors. However, these effects resolved spontaneously over time.

This study had obvious limitation that we would like to acknowledge. First of all, the success rate in both groups were unexpectedly lower than assumed for sample size calculation resulting the reduction in power. Second, we compared the recommended dose of meloxicam with a clinically safe dose of paracetamol, but we do not know whether the two doses compared are equipotent. Third, while the researcher collecting the data remained blinded to the groups ‘assignment the owners were not blinded to the treatment; due to the different dosing intervals of drugs, it would have been impossible not to inform them properly. Fourth, we did not collect or analyse any blood samples during post-operative period, so we can comment only on the safety of these drugs based on clinical signs. However, other studies demonstrated clinically nonsignificant changes after administration of paracetamol to dogs [12, 28]. Finally, the ad hoc questionnaire that we used to identify the need of rescue analgesia was not validated; our results must be considered as first attempt to establish a systematic pain assessment process at home.

## Conclusions

The non-inferiority of paracetamol to meloxicam for the postoperative analgesia after ovariohysterectomy in dogs was not confirmed during the first six hours. In our study, several dogs needed rescue analgesia in both groups during two post-operative days highlighting that frequent assessment of pain is mandatory to ensure efficient analgesia both in hospital and at home. Rescue analgesia might be needed more frequently if paracetamol instead of meloxicam is selected as a post-operative analgesic. Both paracetamol and meloxicam were well tolerated, and only mild self-limiting gastrointestinal side effects were detected.

## Supplementary Information

Below is the link to the electronic supplementary material.

Supplementary Material 1: Additional file 1. Consort flow diagram of the recruitment process. Information is presented as PDF format.

Supplementary Material 2: Additional file 2. The coding of the target force of the von Frey Filament in grams. Information is presented as PFD format.

Supplementary Material 3: Additional file 3. Questions related to general well-being of the dog. Information is presented as PDF format.

Supplementary Material 4: Additional file 4. A modified version of the short form of Glascow Composite Pain Scale. Information is presented as PDF format.

## Data Availability

The datasets used and/or analysed during the current study are available from the corresponding author on reasonable request.

## Declarations

### Prior publication

Data have not been published previously.
