# Supplementary material for: Analgesic and clinical side effects of paracetamol and meloxicam in female dogs after elective ovariectomy or ovariohysterectomy – randomized clinical trial
Source: Acta Vet Scand. 2026 Nov 10;68:50. doi: 10.1186/s13028-026-00892-3 (PMC13753368; doi:10.1186/s13028-026-00892-3)
Supplement: Supplementary file 1 [file 13028_2026_892_MOESM1_ESM.pdf]

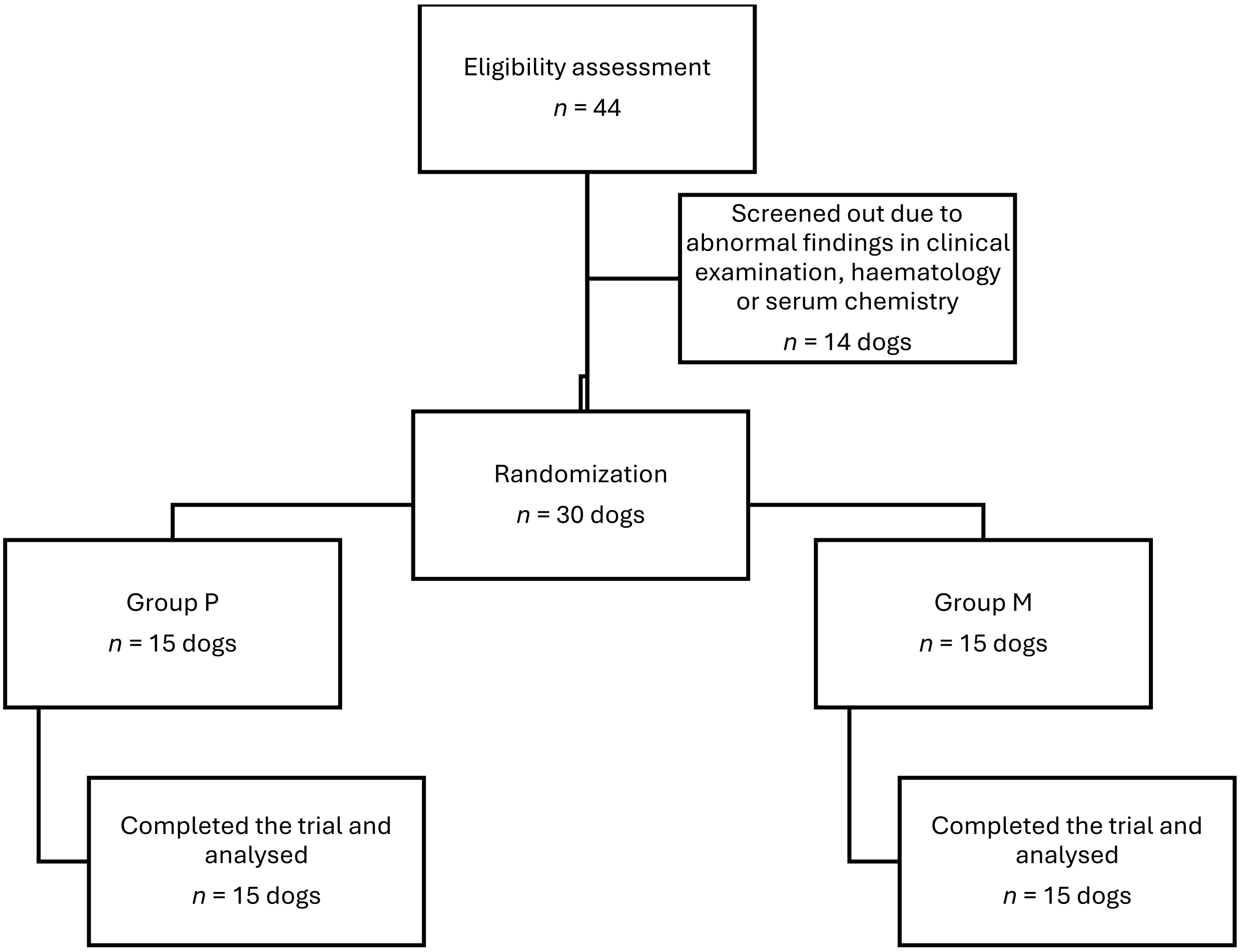
Eligibility assessment
n = 44
Screened out due to abnormal findings in clinical examination, haematology or serum chemistry
n = 14 dogs
Randomization
n = 30 dogs
Group P
n = 15 dogs
Group M
n = 15 dogs
Completed the trial and analysed
n = 15 dogs
Completed the trial and analysed
n = 15 dogs
