# Supplementary material for: Analgesic and clinical side effects of paracetamol and meloxicam in female dogs after elective ovariectomy or ovariohysterectomy – randomized clinical trial
Source: Acta Vet Scand. 2026 Nov 10;68:50. doi: 10.1186/s13028-026-00892-3 (PMC13753368; doi:10.1186/s13028-026-00892-3)
Supplement: Supplementary file 2 [file 13028_2026_892_MOESM2_ESM.pdf]

Code	Target force of the filament (g)
0	< 1
1	1-2
2	4-6
3	8-10
4	15-26
5	100
6	180
7	300
8	No response
