# Supplementary material for: Analgesic and clinical side effects of paracetamol and meloxicam in female dogs after elective ovariectomy or ovariohysterectomy – randomized clinical trial
Source: Acta Vet Scand. 2026 Nov 10;68:50. doi: 10.1186/s13028-026-00892-3 (PMC13753368; doi:10.1186/s13028-026-00892-3)
Supplement: Supplementary file 3 [file 13028_2026_892_MOESM3_ESM.pdf]

Additional File 3

Questions related to general well-being that were presented to owners of the dogs one and two days after elective sterilization surgery. Questions were originally presented in Finnish and translated to English for publication.

**QUESTION**	**YES**	**NO**
Has the dog been drooling?		
Has the dog dysorexia/anorexia?		
Has the dog polydipsia/oligodipsia?		
Has the dog vomited?		
Has the dog polyuria/oligouria?		
Has the fecal consistency been normal?		
