# Supplementary material for: Analgesic and clinical side effects of paracetamol and meloxicam in female dogs after elective ovariectomy or ovariohysterectomy – randomized clinical trial
Source: Acta Vet Scand. 2026 Nov 10;68:50. doi: 10.1186/s13028-026-00892-3 (PMC13753368; doi:10.1186/s13028-026-00892-3)
Supplement: Supplementary file 4 [file 13028_2026_892_MOESM4_ESM.pdf]

Additional File 4. A modified version of the short form of Glascow Composite Pain Scale

1. Look at the dog from distance

Is the dog?

a)	Quiet	0
b)	Crying or whimpering	1
c)	Groaning	2
d)	Screaming	3

Is the dog?

a)	Ignoring any wound	0
b)	Looking at wound	1
c)	Licking wound	2
d)	Rubbing wound	3
e)	Chewing wound	4

2. When you put lead on dog and take it out

When the dog rises/walks is it?

a)	Normal	0
b)	Slower than normal	1
c)	Clearly slow or reluctant	2
d)	Stiff	3
e)	It refuses to move	4

3. Overall

Is the dog?

a)	Happy and content or happy and bouncy	0
b)	Quiet	1
c)	Non-responsive to surroundings	2
d)	Nervous or anxious or fearful	3
e)	Depressed or non-responsive to stimulation	4
